# Understanding the relationship between family income and conduct problems: findings from the mental health of children and young people survey

**DOI:** 10.1017/S0033291722000654

**Published:** 2023-07

**Authors:** P. J. Piotrowska, C. B. Stride, B. Maughan, T. Ford, N. A. McIntyre, R. Rowe

**Affiliations:** 1 Department of Neuroscience, Psychology, and Behaviour, College of Life Sciences, University of Leicester, Leicester LE1 7RH, UK; 2 Management School, University of Sheffield, Conduit Road, Sheffield S10 1FL, UK; 3 Institute of Psychiatry, Psychology and Neuroscience, King's College London, 16 De Crespigny Park, Camberwell, London SE5 8AF, UK; 4 Department of Psychiatry, University of Cambridge, Robinson Way, Cambridge CB2 0SZ, UK; 5 School of Education, University of Southampton, University Road, Southampton SO17 1BJ, UK; 6 Department of Psychology, University of Sheffield, Cathedral Court, 1 Vicar Lane, Sheffield S1 2LT, UK

**Keywords:** socio-economic status, income, conduct problems, family functioning

## Abstract

**Background:**

Children from low-socioeconomic backgrounds exhibit more behavioural difficulties than those from more affluent families. Influential theoretical models specify family stress and child characteristics as mediating this effect. These accounts, however, have often been based on cross-sectional data or longitudinal analyses that do not capture all potential pathways, and therefore may not provide good policy guidance.

**Methods:**

In a UK representative sample of 2399 children aged 5–15, we tested mediation of the effect of household income on parent and teacher reports of conduct problems (CP) via unhealthy family functioning, poor parental mental health, stressful life events, child physical health and reading ability. We applied cross-lagged longitudinal mediation models which allowed for testing of reciprocal effects whereby the hypothesised mediators were modelled as outcomes as well as predictors of CP.

**Results:**

We found the predicted significant longitudinal effect of income on CP, but no evidence that it was mediated by the child and family factors included in the study. Instead, we found significant indirect paths from income to parental mental health, child physical health and stressful life events that were transmitted via child CP.

**Conclusion:**

The results confirm that income is associated with change in CP but do not support models that suggest this effect is transmitted via unhealthy family functioning, parental mental health, child physical health, stressful life events or reading difficulties. Instead, the results highlight that child CP may be a mediator of social inequalities in family psychosocial functioning.

Conduct problems (CP) in young people present a common (Vasileva, Graf, Reinelt, Petermann, & Petermann, 2021) and serious problem for children, their families and society. Children with CP face substantially increased risk of future emotional and behavioural problems, substance abuse and psychosocial difficulties including lower academic achievement (Erskine et al., 2016; Lichtenstein et al., 2020). Understanding the factors that increase the risk for CP is crucial for developing interventions and making policy decisions that can reduce CP to benefit both individual and societal wellbeing.

Lower family social status, whether indexed by income or broader measures of socio-economic status (SES) such as parental education, or occupational status, has been proposed as a causal factor in a number of models of the development of CP in young people (e.g. Conger, Martin, Masarik, Widaman, & Donnellan, 2015). A wealth of correlational evidence supports this relationship. A meta-analysis based on 139 independent estimates from studies published between 1960 and 2012 (Piotrowska, Stride, Croft, & Rowe, 2015) found a mean weighted effect size of −0.10 (95% confidence interval −0.08 to −0.12). Although modest, this confirmed that income was negatively associated with CP in children. Reviews of quasi-experimental studies (Jaffee, Strait, & Odgers, 2012; Maughan, Rowe, & Murray, 2017) further support a causal effect of family SES on offspring CP. Understanding this relationship could have important implications for policy and practice; if there is a causal effect of SES on CP then interventions to improve SES, or to disrupt the pathway between SES and CP, could reduce overall levels of CP and also help to flatten the social gradient in CP (Piotrowska, Stride, Maughan, Goodman, & Rowe, 2015).

SES is often modelled as a distal cause of CP, with more proximal factors mediating its effect. One of the most influential models, the Family Stress Model (FSM; Masarik & Conger, 2017), proposes that economic pressure contributes to parental distress, which in turn impacts upon parenting practices that then increase child CP. A consistent body of work indicates that SES is associated with parental emotional problems, lack of warmth, harsh discipline and poorer home environment quality, and that these factors in turn lead to behavioural problems in both boys and girls (Conger et al., 1992; Dodge, Pettit, & Bates, 1994; Votruba-Drzal, 2006). Other potential intervening variables include child language ability and neighbourhood deprivation (Petersen et al., 2013; Vernon-Feagans, Garrett-Peters, Willoughby, Mills-Koonce, & Family Life Project, 2012). Our previous cross-sectional analyses highlighted unhealthy family functioning, neighbourhood disadvantage, stressful life events and children's literacy difficulties as candidate mediators of the effect of income on CP (Piotrowska, Stride, Maughan, & Rowe, 2019). Quasi-experimental evidence supports the aspects of the FSM and similar models, including showing that increased income improves parental relationships (Akee, Copeland, Costello, & Simeonova, 2018), and that harsh discipline, divorce, parental psychopathology and peer deviance have causal effects on CP (Jaffee et al., 2012). However, these studies have not yet addressed the full mediation pathway from income to family stress and from family stress to CP.

Much of the evidence directly addressing mediation of SES effects on CP is cross-sectional (e.g. Barrera et al., 2002; Conger et al., 2002) and therefore vulnerable to confounding and mis-specification of causal direction. Longitudinal designs with repeated observations of risk factors, mediators and outcomes provide an opportunity to test some of these hypothesised pathways more rigorously, for example, through using a ‘cross-lagged’ approach that can model the direction and stability of effects, and examine the role of change in the mediation process (Preacher, 2015). However, few longitudinal studies addressing potential mediators linking SES and CP have adopted these methods. In particular, few studies have controlled for prior levels of outcome variables when predicting them across time or explored possible reciprocal pathways. For example, from the Family Transitions Project dataset that has provided much of the support for the FSM, Conger et al. (2015) modelled parenting (termed emotional investment) as predicting aggressive behaviour 4 years later in a sample of male and female adolescents (no sex differences were found). But their model did not include aggressive behaviour measured contemporaneously with emotional investment, so could not test whether emotional investment predicted *change* in aggression over time, or whether child aggression may lead to lower parental emotional investment rather than vice versa. This is a potentially important omission as reciprocal longitudinal relationships between family stress and child CP have been reported elsewhere (Choe & Zimmerman, 2014; Serbin, Kingdon, Ruttle, & Stack, 2015).

The present study tests five potential mediators of the effect of family SES on child and adolescent CP in the longitudinal Mental Health of Children and Young People (MHCYP) 1999 survey. We investigate the roles of parental mental health and family functioning that are specified as potential mediators in the FSM, as well as child physical health, stressful life events and reading ability. Equivalent measures of SES, CP and all mediators were collected at baseline and at a 3-year follow-up, allowing estimation of longitudinal cross-lagged effects. Based on our literature review, we expected the hypothesised mediators to yield significant indirect effects that would explain the relationship between income and CP. Finally, we also explore whether any mechanisms found in the study differ by child's age, as different causal mechanisms have been posited for antisocial behaviour that begins during childhood and during adolescence (e.g. Moffitt, 2018).

## Methods

### Sample

Our dataset comes from the MHCYP survey that was carried out by the UK Office for National Statistics in 1999 taking a representative sample of children aged 5–15 years from the general population of England, Scotland and Wales (Meltzer, Gatward, Corbin, Goodman, & Ford, 2000). The procedures are fully described elsewhere; all survey procedures received multi-centre ethics approval (Ford, Goodman, & Meltzer, 2003). The sampling framework identified 12 529 children. At least one of child, parent or teacher response data were collected for 10 438 children (83% of target sample). The 36-month follow-up data were collected in 2002; all children identified as having a psychiatric disorder at *t*_1_ and a random third of children without a disorder at *t*_1_ were targeted. This yielded a *t*_2_ sample of 2938 eligible children, from whom 2586 (88%) completed the follow-up survey. Having matched the *t*_1_ and *t*_2_ samples, 187 cases were removed from the original datasets due to baseline and follow-up incompatibilities such as different parental informant between the two waves (*n* = 158) or an impossible age difference greater than 3 years. The analysis sample for our study was restricted to children for whom parent- or teacher-reported data were available at both time points (*t*_1_, *t*_2_), providing an analysis sample of 2399 (52% boys; mean age at first contact = 9.93 years, s.d. = 3.11). All measures specified below were completed at both time points.

### Measures

#### Conduct problems

Teacher-rated CP were assessed using the Development and Well-Being Assessment (DAWBA; Goodman, Ford, Richards, Gatward, & Meltzer, 2000). Symptoms were assessed on a three-point Likert response scale: not true (0), partly true (1) and certainly true (2). Four behaviours (uses weapons when fighting, deliberately cruel to animals, sets fires deliberately and unwanted sexual activity) were dropped because fewer than 2% of children were reported to engage in these activities. The remaining six items included starting fights, bullying, physical cruelty, lying or cheating, stealing, and vandalism. Parent report of their child's CP was gathered using the five-item CP scale of the Strengths and Difficulties Questionnaire (SDQ; Goodman, 1997) where items are rated on a three-point scale; 0 (not true) to 2 (certainly true).

#### Household income

Caregivers indicated their gross annual household income on a 22-point ordinal scale; the values ranged from ‘no source of income’ (0), ‘less than £1000’ (1), ‘£1000–£1999’ (2), etc., through to ‘over £40000’ (21). The mid-points of the category bands were taken as the income value for respondents within each category, and the measure treated as a continuous scale. Those reporting the highest category (over £40 000) were given the median income for UK citizens earning over £40 000 at the time of the study, which was £48 500.

#### Parental mental health

Parents completed the 12-item General Health Questionnaire (GHQ-12; Goldberg & Williams, 1988) screen for non-psychotic psychiatric disorders which assesses whether recent problems in everyday functioning, such as concentration and sleep problems, are present (1) or absent (0). The GHQ-12 demonstrates good sensitivity and specificity in identifying clinical cases (Goldberg et al., 1997).

#### Family functioning

Parents completed the 12-item General Functioning Scale of the McMaster Family Assessment Device (Epstein, Baldwin, & Bishop, 1983) which assesses decision making, feelings of acceptance and discussions of emotions within the family. Each item is scored strongly agree (1), agree (2), disagree (3) or strongly disagree (4). This scale demonstrates good internal reliability and criterion validity in distinguishing between healthy functioning families from those attending a psychiatric service (Kabacoff, Miller, Bishop, Epstein, & Keitner, 1990).

#### Stressful life events

Parents completed a 10-item scale addressing events such as serious illness of a parent or marital difficulties (Meltzer, Gatward, Corbin, Goodman, & Ford, 2003). For our analyses, we only included items assessed at both initial contact and follow-up. One question addressing parent police contact was also dropped from all analyses as it might reflect the intergenerational transmission of CP (Besemer, Ahmad, Hinshaw, & Farrington, 2017; Meyer et al., 2000). The remaining items assessed separation and marital difficulties, major financial crisis, serious illness/stay at hospital and serious accident. Each item was scored as present (1) or absent (0) and summed to a total score.

#### Physical health and reading

Parents answered ‘How is your child's health in general?’ on a scale from very good (1) to very bad (5). Teachers assessed reading compared with peers as above average (1), average (2), some difficulty (3) or marked difficulty (4).

### Data analysis

Mplus v7.2 was used for analyses (Muthén & Muthén, 1998–2012). SDQ and DAWBA responses were treated as ordered categorical indicators and models were fitted using weighted least square parameter estimates. Analyses were weighted to account for the under-sampling of participants who had not met the criteria for diagnosis at *t*_1_. This allowed results to be generalised to the original nationally representative population.

Data analysis comprised three stages. First, a series of confirmatory factor analyses (CFA) was performed to confirm the structure of the multi-item scales measuring children's CP (as measured by teachers and parents), parental mental health and family functioning and to test factorial invariance between the two time points. Of the remaining mediators, physical health and reading were single items, and the stressful life events measure counted events, so these were omitted from the CFA.

**Fig. 1.**
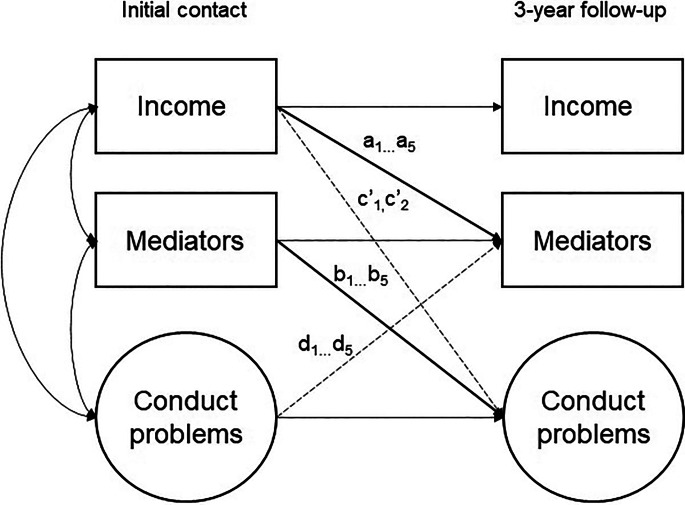
Conceptual diagram of the cross-lagged panel mediation model. *Note:* Subscripted pathways (a, b, d) indicate different coefficients estimated for each of the five mediators. For the purposes of illustrating our conceptual model, our multiple mediators are represented in a single box. Please note that our full model tested all of our observed and latent mediators in separate pathways. Two measures representing conduct problems (parent and teacher reports) are analysed in the model simultaneously (i.e. all results are presented separately for parent- and teacher-reported CP). Age and sex were included as covariates but are not shown in the diagram.

Second, we used structural equation modelling to test our mediational hypotheses. As depicted in Fig. 1 we used the mediation modelling approach recommended for longitudinal designs with two measurement occasions to achieve temporal ordering both between predictor measured at *t*_1_ and mediators measured at *t*_2_ and between mediators measured at *t*_1_ and outcomes measured at *t*_2_ (Cole & Maxwell, 2003; Preacher, 2015). Income was treated as a continuous measure, using the category mid-points as described above, and tested as a predictor of each of the five hypothesised mediators (parental mental health and family functioning, stressful life events, child's reading ability and physical health) as measured at *t*_2_ (a paths). Income was also modelled to have a direct effect on the teacher- and parent-reported CP (c’ paths). Each of the mediators at *t*_1_ was modelled to predict the two children's CP outcomes at *t*_2_ (b paths). The parent- and teacher-reported child behaviour outcomes were allowed to correlate. Tests for mediation between income and CP followed the general approach of Hayes (2013) where the indirect path is tested as the product of paths a and b, with confidence intervals estimated using bias-corrected bootstrapping. Indirect effects from income to each CP outcome via each mediator variable were calculated and tested simultaneously.

This approach also allowed the examination of possible ‘reverse causality’ links between CP at *t*_1_ and changes in the hypothesised mediators (e.g. family functioning) at *t*_2_ (d paths). The inclusion of the reverse paths d and the comparison of paths b and d helped to assess the alternative indirect pathways whereby CP was modelled as a mediator of the effect of income, for example, on parental mental health, by assessing the product of paths c and d. Each model also included child age (years) and sex [female (0) and male (1)] as covariates predicting both mediators and outcomes. Finally, a series of multi-group models tested whether mediation parameters (paths a, b, c’, d) differed significantly between age groups (younger: 4–10, older: 11–16). Models allowing these parameters to differ were compared against models fixing them to be equal using the adjusted χ^2^ test.

## Results

At the first analysis stage, the structure and factorial invariance of children's CPs (teacher and parent report), parental mental health and family functioning was tested. The model fixing factor loadings, thresholds and factor variances to be equal across time (i.e. strict temporal invariance) gave a good fit according to Hu and Bentler's criteria (1999) [χ^2^ = 4834.14, df = 2386, comparative fit index (CFI) = 0.972, root mean square error of approximation (RMSEA) = 0.021], and was not weaker than models in which some or all of the loadings, thresholds and variances were allowed to differ across time. Standardised factor loadings from this model (online Supplementary material) show that all items loaded strongly onto their respective factors. This model formed the basis of subsequent analyses.

The established CFA model as described above was extended into the full mediation model by including the predictor variable (i.e. income), the three remaining mediators (i.e. stressful life events, child's reading ability and physical health), the two CP outcomes (parent- and teacher-reported CP), as well as the covariates of age and sex. This model provided a good fit (χ^2^ = 6676.27, df = 3063, CFI = 0.96, RMSEA = 0.022). As expected, boys showed significantly more CP than girls by both parent (*b* = 0.14, 95% CI 0.03–0.24, *p* = 0.013) and teacher (*b* = 1.98, 95% CI 1.23–3.24, *p* = 0.001) report. Parent-reported CP were more common in younger children (*b* = −0.03, 95% CI −0.05 to −0.01, *p* = 0.001) while teacher-reported CP were unrelated to age (*b* = 0.01, 95% CI −0.09 to 0.10, *p* = 0.91). All correlations between income, the mediators and the two antisocial outcomes were significant with the exception of the association between *t*_2_ teacher-reported CP and child physical health at *t*_2_ (Table 1).

**Table 1.** Correlations among income, mediators and CP outcomes at first contact and follow-up

	1.	2.	3.	4.	5.	6.	7.	8.	9.	10.	11.	12.	13.	14.	15.	16.
1. *t*_1_ Income																
2. *t*_2_ Income	0.78***															
3. *t*_1_ Parent report CP	−0.23***	−0.25***														
4. *t*_2_ Parent report CP	−0.23***	−0.23***	0.76***													
5. *t*_1_ Teacher report CP	−0.29***	−0.24***	0.61***	0.53***												
6. *t*_2_ Teacher report CP	−0.23***	−0.18***	0.49***	0.54***	0.62***											
7. *t*_1_ Unhealthy family functioning	−0.13***	−0.15***	0.34***	0.30***	0.16***	0.18***										
8. *t*_2_ Unhealthy family functioning	−0.12***	−0.14***	0.32***	0.41***	0.19***	0.14***	0.65***									
9. *t*_1_ Parental mental health	−0.17***	−0.18***	0.25***	0.23***	0.11**	0.12**	0.32***	0.25***								
10. *t*_2_ Parental mental health	−0.14***	−0.18***	0.28***	0.36***	0.13**	0.17***	0.22***	0.35***	0.49***							
11. *t*_1_ Child physical health	−0.16***	−0.17***	0.21***	0.19***	0.09**	0.09*	0.14***	0.09***	0.19***	0.15***						
12. *t*_2_ Child physical health	−0.17***	−0.19***	0.20***	0.28***	0.21***	0.07	0.20***	0.21***	0.19***	0.21***	0.44***					
13. *t*_1_ Stressful life events	−0.31***	−0.24***	0.19***	0.17***	0.20***	0.15***	0.10***	0.08***	0.26***	0.17***	0.19***	0.16***				
14. *t*_2_ Stressful life events	−0.09***	−0.18***	0.09***	0.15***	0.11***	0.08*	0.07**	0.07**	0.16***	0.19***	0.11***	0.12***	0.18***			
15. *t*_1_ Reading	−0.25***	−0.23***	0.27***	0.29***	0.33***	0.28***	0.12***	0.08**	0.09**	0.07*	0.17***	0.17***	0.12***	0.08***		
16. *t*_2_ Reading	−0.27***	−0.26***	0.35***	0.37***	0.44***	0.39***	0.09**	0.09**	0.07*	0.09*	0.17***	0.21***	0.11***	0.10***	0.68***	

*p < 0.05; **p < 0.01; ***p < 0.001.

When assessing the mediation process from income to CP, we first inspected the paths linking *t*_1_ income to each of the five mediators measured at *t*_2_ (Fig. 1, paths a_1_–a_5_). As Table 2 shows, all coefficients were negative, and those for unhealthy family functioning and child physical health were significant, indicating a negative association between income and adversity.

**Table 2.** Paths from income at initial contact to hypothesised mediators at follow-up

Path from income to mediator	Estimate	95% confidence interval
Unhealthy family functioning	−0.03*	−0.05 to −0.01
Parental mental health	−0.03	−0.08 to 0.02
Child physical health	−0.10**	−0.17 to −0.03
Stressful life events	−0.02	−0.05 to 0.01
Reading	−0.05	−0.12 to 0.02

*p < 0.05; **p < 0.01; ***p < 0.001.

Next, we inspected paths between the mediators measured at initial contact and the parent- and teacher-reported CP follow-up measures (Fig. 1, *b*_1_–*b*_5_ estimated separately for the two CP outcomes), controlling for CP at initial contact (Table 3). Unexpectedly, none of these coefficients were significant, providing no evidence that any of the hypothesised mediators at *t*_1_ were associated with changes in CP between the time points. Indirect effects calculated as the products of paths a and b, and bias-corrected bootstrapped confidence intervals are also presented in Table 3. None of these indirect effects were significant.

**Table 3.** Paths from mediators at *t*_1_ to conduct problems at *t*_2_ and estimated indirect effect of income on conduct problems via each mediator

Outcome	Parent report CP				Teacher report CP			
Mediator	CP prediction	95% CI	Indirect	95% CI	CP prediction	95% CI	Indirect	95% CI
Unhealthy family functioning	0.01	−0.07 to 0.09	<0.001	−0.003 to 0.002	0.33	−0.23 to 0.99	−0.01	−0.03 to 0.01
Parental mental health	0.01	−0.04 to 0.07	<0.001	−0.004 to 0.001	0.15	−0.31 to 0.65	−0.01	−0.04 to 0.01
Child physical health	−0.01	−0.07 to 0.05	0.001	−0.01 to 0.01	−0.05	−0.56 to 0.38	0.01	−0.04 to 0.06
Stressful life events	0.01	−0.04 to 0.06	<0.001	−0.002 to 0.001	−0.10	−0.48 to 0.22	0.002	−0.004 to 0.02
Reading	0.02	−0.03 to 0.07	−0.001	−0.01 to 0.001	0.07	−0.26 to 0.39	−0.004	−0.04 to 0.01

CI, confidence interval; CP, conduct problems.

None of the coefficients are significant at *p* < 0.05

Table 4 shows the ‘reverse causality’ paths (Fig. 1, *d*_1_–*d*_5_) between CP at *t*_1_ and the hypothesised mediators at *t*_2_. As elsewhere in the model, initial levels of the mediators were controlled. Therefore, these paths tested whether CP at the first contact was related to change in the hypothesised mediators between baseline and follow-up.

**Table 4.** Paths from conduct problems at initial contact to the mediators at follow-up

	Parent report CP		Teacher report CP	
	Estimate	95% CI	Estimate	95% CI
Unhealthy family functioning	0.07*	0.02–0.13	0.00	−0.01 to 0.02
Parental mental health	0.32**	0.10–0.56	−0.03	−0.10 to 0.03
Child physical health	0.35**	0.14–0.62	−0.01	−0.08 to 0.05
Stressful life events	0.11**	0.04–0.21	−0.02	−0.05 to 0.004
Reading	−0.05	−0.33 to 0.17	0.11**	0.04–0.21

CI, confidence Interval; CP, conduct problems.

*p < 0.05; **p < 0.01; ***p < 0.001.

The general pattern in Table 4 is that higher CP at first contact is associated with increased adversity (i.e. higher levels of the hypothesised mediators) at follow-up. For parent-reported CP, these associations were significant for unhealthy family functioning, parental mental health, child physical health and stressful life events. Conversely, teacher-reported CP at first contact was only significantly related to worsening reading level between initial contact and follow-up. When the paths from mediators at initial contact to CP at follow-up (b_1_–b_5_) were tested against their respective ‘reverse causality’ pathways from CP at initial contact to mediators at follow-up (d_1_–d_5_), none were significantly different for teacher-reported CP. For parent-reported CP, the links between CP at initial contact and parental mental health, child physical health and stressful life events at follow-up were significantly different from their respective pathways from the hypothesised mediators to CP (all *p*s < 0.05). The confidence intervals for the three indirect effects from income, via CP, to parental mental health [estimate (95% CI) = −0.016 (−0.04 to −0.003)], child physical health [estimate (95% CI) = −0.018 (−0.04 to −0.01)] and stressful life events [estimate (95% CI) = −0.006 (−0.01 to −0.001)] did not include 0 and therefore show they were significant.

The final paths of interest were between income at initial contact and parent- and teacher-reported CP at follow-up (path c’, Fig. 1). These paths model the effect of income on later CP that is independent of the specified mediators. The path to parent-reported CP was significant [estimate (95% CI) = −0.05 (−0.09 to −0.02), *p* = 0.006], indicating that higher income at initial contact was related to lower levels of CP at follow-up, controlling for initial CP levels. The path from income to teacher-reported CP was non-significant [estimate (95% CI) = −0.23 (−0.53 to 0.03), *p* = 0.127] showing no direct effect of income on change in teacher-reported CP. Finally, multi-group models showed that the results were not moderated by child's age. The full model with all mediation paths (a, b, c’, d) fixed to be equal across age groups (younger: 4–10, older: 11–16) did not fit significantly worse than a model allowing these parameters to differ (χ^2^ = 38.45, df = 27, *p* = 0.07).

## Discussion

This study was designed to provide a longitudinal test of five potential mediators of the effect of family SES on child and adolescent CP: unhealthy family functioning, parental mental health, stressful life events, child physical health and reading levels. It also explored the possibility of reciprocal effects and tested equivalence across age. As expected, we found an effect of income on later CP, after controlling for initial CP levels. Unexpectedly, we found no evidence that this effect was transmitted via the hypothesised mediators. Despite some significant effects of income on the intermediate variables (unhealthy family functioning and child physical health), there were no significant effects of these proposed mediators on change in CP. This is contrary to models such as the FSM, which posit that family and parental variables mediate the relationship between SES and CP (Conger, Ge, Elder, Lorenz, & Simons, 1994).

One potential explanation for this discrepancy is that the measures used here captured the concepts of familial wellbeing and child characteristics differently from the approaches used in other studies. However, a previous cross-sectional analysis of the MHCYP study conducted in 2004 (i.e. 5 years after the data analysed here were collected) that used similar measures of income, mediators and antisocial outcomes found that unhealthy family functioning, neighbourhood disadvantage, stressful life events and child's literacy difficulties mediated the effect of SES on CP (Piotrowska et al., 2019). This emphasises the possibility that differences in analytic approach account for the discrepant results; as noted in the introduction, many of the studies on which existing models are based were either cross-sectional or did not control for previous levels of proposed mediators and outcomes – a key feature of the cross-lagged approach used in the present study. Therefore, it seems plausible that the discrepancy results from the more rigorous analytic approach of our current design and emphasises the importance of controlling initial levels of CP and considering alternative developmental pathways.

While our study did not find the expected mediation of the effect of income on CP, we did identify indirect effects of SES on child and family functioning that were mediated via child CP, further emphasising the suitability of our approach for identifying mediation. There were three significant indirect pathways of this kind, running to parental mental health, children's physical health and stressful life events. It is possible that children's disruptive behaviour contributes to family stress, and that over time this affects parental wellbeing. Similarly, children with elevated CP have increased rates of accidents and hospitalisations, often directly or indirectly resulting from their behaviour (Rowe & Maughan, 2009). This could contribute to the effect of CP on physical health and stressful life events observed in this study. An increasing number of studies have identified bidirectional relationships between children's behavioural development and parenting (e.g. Pearl, French, Dumas, Moreland, & Prinz, 2014; Serbin et al., 2015), but no existing studies have tested bidirectional paths as mechanisms underlying the relationship between income, CP and family environment. Considering these reverse causality pathways is crucial as it highlights a range of potential *consequences* of CP for the child and the family, and emphasises the policy importance of reducing child CP in order to tackle social inequalities in family adversity.

The pattern of results differed somewhat between parent- and teacher-reported CP. In the reverse paths from CP at initial contact to family and child variables at follow-up, parent-reported CP significantly predicted higher levels of unhealthy family functioning, poorer parental mental health and child physical health, and more stressful life events while teacher-reported CP only predicted reading difficulties. This is consistent with previous research suggesting the existence of informant-specific effects, and showing that parental distress, family functioning and child physical health problems can be more strongly associated with parent than with teacher ratings of CP (Collishaw, Goodman, Ford, Rabe-Hesketh, & Pickles, 2009). It is unclear whether the effects of this kind reflect rater bias or situational differences in child behaviour. It is likely that teachers are well placed to assess the aspects of CP relevant to academic development in classroom settings whereas parents may be better placed to observe the components of CP most relevant to family functioning. Finally, age did not affect the mediation estimates in the current study. This fits with previous research that has reported similar relationships between income and parenting within families of young children and adolescents (Barajas-Gonzalez & Brooks-Gunn, 2014). This finding suggests that, despite some well-established age differences in CP (e.g. Lahey et al., 2000), mechanisms underlying social inequality in CP may be the same.

Alongside the strengths of the current study – including a nationally representative sample, longitudinal design, simultaneous modelling of multiple mediators and exploration of reverse causality pathways – there were some limitations. Firstly, we were limited in the choice of potential mediating variables by the measures available in MHCYP 1999 at both time points. However, the most commonly studied mechanisms such as parental mental health and family functioning were included. The study is also limited to two time points, although our modelling approach capitalised on the available temporal ordering between both risk and mediators, and between mediators and outcomes.

It is also important to note that the data used in this study were collected in 1999–2002, and it is possible that relationships between CP and income have changed over time. Replication and extension of this work in other, more recent longitudinal studies is of high priority, particularly with regard to modelling bidirectional pathways between SES, ‘intermediate’ mediating factors and CP, for example, in developmental cascade models or random intercepts cross-lagged panel models (RI-CLPM). The RI-CLPM models can partial out between-person variance to ensure that the lagged relationships represent within-person effects (Hamaker, Kuiper, & Grasman, 2015). This approach, however, requires at least three waves of data and as such could not be tested here but should be considered in future studies.

Furthermore, given the significant effects of CP on a range of family variables, it will be important for future studies to consider the impact of children's psychopathology on family financial distress which was beyond the scope of the current study. Several studies have estimated the cost of CP (e.g. Romeo, Knapp, & Scott, 2006), however, to the best of our knowledge, no studies have yet considered the association between income and CP and potential mechanisms suggested by the current study. Finally, it is important to emphasise that our study did identify an effect of income on later CP and it is likely that this is mediated by factors other than those included in our analyses. Therefore, it is important for future studies to include a wider range of mediators such as parenting (e.g. Bornstein, Putnick, & Suwalsky, 2018) and school characteristics (e.g. Higgins, Perra, Jordan, O'Neill, & McCann, 2020).

Identifying the factors that mediate the effect of family SES on child CP remains an important goal to provide intervention targets in order to minimise social inequalities in CP during childhood and adolescence. Our findings also highlight the importance of intervening with CP to improve parental distress, stressful life events and physical health. Parenting programmes offer effective methods to improve CP (Leijten et al., 2019). Evaluation studies often include parental mental health as a potential secondary outcome and some (e.g. Piotrowska et al., 2020), but not all (e.g. Gardner, Burton, & Klimes, 2006) have found that parental internalising symptoms are improved by parenting interventions. Our results provide impetus to test whether interventions for CP are also effective in improving child health and reducing stressful life events. If such effects can be identified then this will further strengthen the priority of reducing CP in young people.

## Supplementary material

For supplementary material accompanying this paper visit https://doi.org/10.1017/S0033291722000654. click here to view supplementary material
