# Supplementary material for: Understanding the relationship between family income and conduct problems: findings from the mental health of children and young people survey
Source: Psychol Med. 2022 Mar 21;53(9):3987–94. doi: 10.1017/S0033291722000654 (PMC10317806; doi:10.1017/S0033291722000654)
Supplement: Supplementary file 1 [file S0033291722000654sup001.docx]

**Online Supplement**

Standardised factor loadings in the measurement invariant CFA model

| **Item description** | **Estimate** |
| --- | --- |
| *Teacher-rated conduct problems* |  |
| Starting fights | 0.948 |
| Bullying | 0.893 |
| Physical cruelty | 0.862 |
| Lying or cheating | 0.881 |
| Stealing | 0.849 |
| Vandalism | 0.828 |
|  |  |
| *Parent-rated conduct problems* |  |
| Temper tantrums | 0.639 |
| Generally obedient | 0.703 |
| Fighting, bullying | 0.771 |
| Lying or cheating | 0.761 |
| Stealing | 0.776 |
|  |  |
| *Parental mental health* |  |
| Able to concentrate | 0.749 |
| Losing sleep over worry | 0.754 |
| Playing useful part in things | 0.760 |
| Capable of making decisions | 0.818 |
| Constantly under strain | 0.804 |
| Could not overcome difficulties | 0.890 |
| Able to enjoy normal activities | 0.833 |
| Able to face up to problems | 0.881 |
| Feeling unhappy and depressed | 0.913 |
| Losing confidence | 0.864 |
| Thinking of yourself as worthless | 0.866 |
| Feeling reasonably happy | 0.908 |
|  |  |
| *Family functioning* |  |
| Planning family activities is difficult | 0.610 |
| Can turn to each other for support | 0.585 |
| Cannot talk to each other about sadness | 0.674 |
| Individuals are accepted for what they are | 0.525 |
| Avoid discussing fears and concerns | 0.718 |
| Can express feelings to each other | 0.714 |
| Lots of bad feeling in the family | 0.783 |
| Feel accepted for what we are | 0.672 |
| Making decisions is a problem in our family | 0.693 |
| Able to make decisions on how to solve problems | 0.650 |
| Do not get along well together | 0.746 |
| Confide in each other | 0.694 |
